# Occupational therapy students experience professional reasoning during their practice-based learning: A dialogical analysis

**DOI:** 10.1177/03080226251326518

**Published:** 2025-03-20

**Authors:** Clare Beanlands, Nicki Thomas, Lynn Summerfield-Mann, Sarah Church

**Affiliations:** 1 London South Bank University, London, UK; 2 AECC, University College Bournemouth, Bournemouth, UK

**Keywords:** Occupational therapy student, practice-based learning, professional reasoning, professional identity, dialogical, emotion

## Abstract

**Introduction:**

Professional reasoning is important in making informed and autonomous decisions and practice-based learning provides an opportunity for students to develop this. This study explores the experience of professional reasoning during practice-based learning from the student’s perspective.

**Method:**

A dialogical approach was used, and this is a qualitative approach rooted in the analysis of dialogue and subjectivity. It is based on the philosophy of Mikhail Bakhtin. Bakhtin suggests speech is composed of different genres indicating how we position ourselves in relation to others. Twelve occupational therapy students were interviewed after their final practice placement.

**Findings:**

Professional reasoning during practice-based learning was experienced as complex, multifactorial and contextually influenced. It was a means of enacting, affirming, and negotiating a professional identity, and of being socialised into the reasoning of the profession. Person and occupation-centred values, narrative and empathy influenced students’ reasoning but could conflict with educator and institutional demands. The findings also illustrate the emotionality of learning to reason.

**Conclusion:**

Both the cognitive and affective aspects of learning to reason need consideration when supporting students. Educators and universities need to address the emotionality associated with reasoning and support students to develop person and occupation-centred practice within the pragmatic constraints of practice.

## Introduction

Professional reasoning is defined as ‘the processes used by practitioners to plan direct and perform and reflect on client care’ (Boyt-Schell and Schell, 2025: 4). The World Federation of Occupational Therapists (2016) identifies it as a key competency underpinning entry to the profession. Professional reasoning skills are important in making autonomous, informed decisions that can be justified and defended (Health and Care Professions Council, 2023). The term professional reasoning is considered more inclusive of non-medical practice settings (Boyt-Schell and Schell, 2025); otherwise, the terms clinical and professional reasoning have been used synonymously.

Practice-based learning allows students to navigate the complex task of developing their professional reasoning skills. This study aimed to explore how occupational therapy students experienced professional reasoning during their practice-based learning.

## Literature review

A substantial body of literature addresses educational approaches aimed at developing professional reasoning during students’ university-based studies. Henderson et al. (2017) identify 27 different instructional methods used to develop professional reasoning. By contrast, reasoning during practice-based learning has received little attention. Two studies examine blogging to develop reasoning during practice-based learning. Newton-Scanlan and Hancock (2010) found a format to structure online postings significantly increased students’ reasoning. However, Wild et al.’s (2013) survey found only 27% of students agreed that blogging developed reasoning. This suggests that whilst blogging can develop reasoning, it has limited acceptability. Summerfield-Mann (2010) examined occupational therapy students’ reasoning in a placement case study. She concluded that the practice learning environment was important and that reasoning is individual, complex and socially situated. These findings are comparable to reviews of occupational therapists’ reasoning. Da Silva Araujo et al. (2022) identified the dynamic, complex and contextual nature of reasoning. The multiple modes of reasoning occupational therapists use have been identified (Berndt et al., 2022). DuBroc and Pickens (2015) suggest that expert reasoning is more client-centred and contextually informed. Occupational therapists integrate top-down (occupation-focussed) reasoning and bottom-up (impairment-focussed) reasoning (Hess and Ranmugondo, 2014; Lam Wai Shun et al., 2021). Contextual factors influence reasoning, and the institutional context can restrict reasoning (Carrier et al., 2020). Together, these studies demonstrate the complex, dynamic and multi-layered nature of occupational therapists’ professional reasoning.

This study aimed to explore the experience of professional reasoning during practice-based learning and to understand this phenomenon from the student’s perspective.

## Method

### Methodology

The choice of methodology was influenced epistemologically by post-structuralism and social constructivism. A dialogical approach (Sullivan, 2012) was used, and this is a qualitative approach rooted in the analysis of dialogue and subjectivity. It is based on the philosophy of Mikhail Bakhtin, a philosopher, social thinker and literary critic. Dialogism resists finalisation, values multiple views (polyphony) and understands speech as being composed generically (Bakhtin, 1986). A *genre* is ‘a loose set of stylistic conventions’ (Sullivan, 2012: 45) conveying a perception of reality. For example, reality could be perceived as a romantic quest, an epic struggle or a transformative adventure. A dialogical approach is useful for research questions associated with the relationship between contexts, peoples’ experiences and how their world of meaning is felt and thought through (Sullivan, 2012), making it suitable for exploration of students’ reasoning in the practice learning context. Although a relatively new approach, it is being used by researchers in both health and social care and education (e.g. Madhill and Sullivan, 2010; Cunningham, 2016).

### Ethics

This study received ethical approval (reference number HSCSEP17/15) and informed written consent was gained from all participants. The first author who conducted the study was known to the students as a lecturer but was not involved in the assessment of the cohorts of students invited to participate in the study. Data were de-identified and stored on a password-protected computer. To preserve anonymity participants were identified by a gender-neutral pseudonym and no information was gathered regarding their characteristics or the placement setting. The first author used a reflexive diary throughout the study.

### Participants

All final year students who had completed all their placements were invited to participate. Twelve students were recruited.

### Data collection

The first author completed an unstructured interview with each student after their final placement. An unstructured interview was selected as it allowed the participants more influence over what was discussed than a semi-structured interview. The participants could choose what to include and how to structure the narration of their experiences, rather than the researcher’s questions structuring the interview. The interview began with the prompt ‘Can you tell me about professional reasoning during your placements, all the events and experiences that were important for you?’ On average, the interviews lasted 90 minutes, they were conducted, audio-recorded and transcribed by the first author.

The reflexive journal assisted in recognising the role of the researcher in co-construction of data, for example, a researcher prompt potentially imposed structure and influenced the participant’s narration in relation to time and space. This type of prompt was avoided in subsequent interviews.

### Data analysis

Data analysis used Sullivan’s (2012) dialogical approach. This approach keeps stories whole rather than dissecting them into codes and themes. The process involved.

• Familiarisation with the data (multiple readings of transcripts).
• Identification of key moments (a key data extract and means of data reduction). Key moments needed to meet four criteria.
 • An experience of reasoning during practice-based learning.
 • A focus on lived truth rather than abstract truth.
 • Emotional investment from participant.
 • Demands a response from the listener.

Fifty-seven key moments were selected and analysed for the genre. Genres were identified by paying attention to emotional intonation, perception of time and space and discourse (e.g. use of parody). The first author selected the key moments, to improve rigour the second and third authors reviewed one transcript with the identified key moments and the emerging genres.

Each key moment was summarised in a table. Table 1 provides examples of some key moments.

- The final step involves writing about the genres.

**Table 1.** Examples of key moments selected for analysis.

Participant	Genres and discourse	Emotional register (of learning/truth)	Time-space elaboration	Context
Alex	Professional, romantic	Empathy, pride, passion, satisfaction, disappointment, determination	Thinking quickly on the spot. Chronological time interrupted by misfortunes. Future-orientated – got to have a product	Working with service user to develop cooking skills
Charlie	Bildungsroman, professional	Illumination	Future orientation. Moment of realisation (light bulb). Risk biggest thing	Service users with amputation and steps reasoning around acceptable risk
Chris	Romantic, double-voiced discourse, irony, professional	Empathy, frustration – with educator, beneficence	Future orientation – the type of therapist I want to be. Family forward-thinking re spouse’s future surgery. Educator here and now – just here for my patient. Student future-orientated preventative	Educator does not explain reasoning to service user
Eli	Romantic, professional genre	Empathy, passion, confidence, satisfaction/achievement	Face immediately lit up. Ran over to the cupboard. All of a sudden, back on track. A little bit of a moment where occupational therapists are quite special. An a-hah moment, all reasoning came together. Without educator pressure off, time to reason. Educator butting in – just needed more time	Student does not understand educator’s reasoning for non-occupation-based intervention so introduces occupation-focussed intervention
	Carnival, romantic, loophole, professional, bildungsroman	Impulsivity, empathy, irreverence, passion, illumination	Reasoning is not so in the moment. Very much after. Chopping and changing. Service users reasoning alongside them. How little time their educator needs to reason compared to them. All coming from reasoning	Spontaneous decision to let service user chop vegetables becomes risky
	Adventure novel, parody, professional	Overwhelmed, independence, openness, determination resilience, pride in achievement	Lack of space and time. Bootcamp in SOAP notes. Get into an educator’s mindset. Now not using the educator’s approach. Analysis bringing it together. Plan what’s the next step	SOAP notes bootcamp – documenting reasoning in an acute setting
	Bildungsroman, travel	Anxiety (being watched), relief (not being watched), frustration (PT/OT butting in)	Hierarchy of cognition. The pressures off. Eventually, get there. Took me a little bit of time. Butting in	Time to reason – hierarchy of cognition
	Bildungsroman, professional	Relief (time and space), amazement/wonder (can reason in a moment), confidence	Building rapport. Barriers came down. Space and time. Pressure. In moment. Very much after. On autopilot. Dipping toe in. Reasoning comes on top (big thing). Not quite there	Reasoning at the moment querying service user’s cognition, had space and time to reason
Frankie	Bildungsroman, professional	Challenged, amazement and wonder, pride	Nine-track mind. Learning reasoning all the time. Loads of different modes of reasoning	Nine-track reasoning – the complexity of reasoning
	Adventure	Sad, disappointment, determination, challenged, isolation, positivity, openness	Newness of setting. Isolation. Buckled down. Speed – needs to be faster. Forward movement	SOAP notes bootcamp needed to copy educator
Jordan	Travel, professional	Stifled, disorientated, challenged	Less time for narrative in acute. Time for narrative in the community. Back to baseline	Lack of opportunity to develop a relationship with patient stifles reasoning
	Bildungsroman	Challenged, determination/resilience, persistent (initially), supported	Squeeze thinking into the model. In-depth. Shape and structure thinking	Use of a model to structure thinking
Jules	Carnival, bildungsroman, super addressee	Independence, loss of control, unrestricted	Could not finish. Completely out of my control. Less formal, less structured. Trying not to restrict. Distance between then and now. Took the sheet off me	Completing an OCAIRS (Occupational Circumstances Interview Assessment and Rating Scale) assessment with service user
	Romance, professional, irony	Reassured, passion	Understanding of occupation solidifies the mind. How to pick apart. Always had a starting point	Occupation starting point for reasoning
Logan	Bildungsroman, professional	Support	Chronological. Note writing allowed time for reflection	SOAP notes
Morgan	Travel	Overwhelmed, pressured	They were so fast. Provide intervention there and then. Miss out things	Difficulty in following experienced therapists’ reasoning
Rowan	Travel	Overwhelmed, disorientation, challenged, dependent, unsupported	Fast – no time to think. What’s next. The gap between theory and practice	Acute setting needs fast-paced reasoning
	Romantic, professional, sideways glance/loophole, irony	Beneficence, empathy	Roll it out. Educator – narrow thinking. Student broader. Students have more empathy, more aware	Student’s person-centred values clash with educator/context
Sam	Bildungsroman, professional	Confusion, overwhelmed, determination/resilience, illumination	Swamp – difficult to navigate a way out. Stepping back. Resolve with time. Swimming in swamp is an important part of the process. Becomes automatic, strange swamp was there. Alien – where are they getting that from	Complexity of reasoning. Repetition helpful
	Travel, bildungsroman, romantic, professional	Challenged, disorientation, passion, confidence, resignation, beneficence	Distance ‘gulf’, ‘split’, ‘divorced’. Reductionist. With people	Experience of an acute hospital context and how they had to reason what sort of therapist they wanted to be
Val	Travel, professional, romantic, bildungsroman	Embarrassment, anxious, overwhelmed, pressured, empathy, supported	Tripped up. Assessment not linear. Moved closer. Too much for my brain. Where are we going	Completing a first initial assessment in the service user’s home
	Romantic, professional	Passion, satisfaction, empathy	Back to see them a lot of times. The service user did not leave the house. Went shopping by themselves	Service user barely leaves home, student helps them participate in more occupations

SOAP: subjective, objective, analysis, plan.

## Findings

The findings are organised according to the genres identified through analysis of the participant’s transcripts. Two major genres (Bildungsroman and romance) were identified in all the transcripts. Three minor genres (travel, adventure and carnival) were also identified in several of the transcripts. The professional genre was also referred to fleetingly by participants for example, when using the professional language of an occupational therapist. Given it was not as predominant as the other genres it is not discussed in this article.

### Bildungsroman

Bildungsroman is a German word meaning novel of development (Michelson, 2012). It is a genre that focuses on an individual in the process of becoming, with the world experienced as a school, and idealism of youth becoming resigned to mature pragmatism (Bakhtin, 1986). It was used by participants to explain the maturation and development of their reasoning.

Participants developed an understanding of the complexity of reasoning:

I don’t even think it’s a three-track mind, I think it’s like a nine-track mind [both laugh] . . .it’s not just, I’m going to use procedural for this, I’m going to use interactive. . . you’re using everything all at the same time. (Frankie)

This complexity meant reasoning could be overwhelming:

Being in the swamp and then just swimming around and being like I don’t know how to make sense of it. . .Educators may vary it to give you variety, but then it is hard to put together the steps or the structure to make sense of what is going on. (Sam)

Autonomy and time and space helped the development of reasoning:

My educator kind of gave me a bit of breathing space. . .I felt really kind of safe to explore things and read things and try things out. (Eli)

Scaffolds such as documentation guidelines supported reasoning:

I found the note writing really helpful. . .in working through my clinical reasoning. . . using the analysis part to analyse what had happened in the session. (Logan)

Some participants found models helped structure reasoning:

(They) really used a specific model. Initially. . . I felt like I had to try and squeeze my thinking into those into those aspects. . .as I, thought more in that way, it actually became easier. . .it did actually help you to put together a picture. . .to get much more of a structure to my thinking. (Jordan)

The practice educator discussing their reasoning could be illuminating. Charlie’s discussion of a young amputee managing a step and Val’s of road safety illustrate this:

A bit of a light bulb moment. . . around, managing risk. . . .advice wasn’t given to them to not do that. . . .having that discussion with my educator. . ., it’s not the safest thing, . . .But. . .being young, they want their independence. . ., that makes absolute perfect sense. (Charlie)

. . .hard not to be a little bit risk adverse. . . The OT was saying. . .it was fine and all the other times they did look. . . I was a bit like ‘oh God they didn’t look one particular time’ and quite nervous about that. (Val)

### Romance

In this genre, the individual’s actions are determined by their values (Bakhtin, 1990). Integrity is tested, and identity is established and affirmed (Bakhtin, 1981). Participants used this genre to discuss the person-centred and occupation-centred values informing their reasoning and their developing professional identity.

Occupation was a foundation for reasoning:

How does it impact your ability to engage in occupation?. . .that applies in any situation. . .and I always had a starting point. I was so comforted to know that. (Jules)

Some participants found the values informing their reasoning differed from their educators:

It felt very kind almost robotic (laughs) I was a bit concerned by the lack of sort of person-centred. . . like conditional reasoning. This patient had lots of other things going on, . . .is there anything else we can do other than just send them home with a bath board?. . . They were much more narrow thinking whereas my perspective on things was a lot broader. Being a student, I guess, like feeling that more kind of empathy. (Rowan)

They weren’t explaining that to the family and reasoning it to them in a way that they understood. . .In the end, they just refused it because (laughs) my educator didn’t explain the reasoning . . .If they say it, it’s not necessarily the right way to do it. It probably made me think, . . .I do have my own mind to have my own thoughts and reasoning. (Chris)

Person-centred and occupation-centred values informed participants’ reasoning around interventions. Val thoughtfully considered occupations that would engage a service user:

Just sat in their own day after day. . . trying to find. . . something that they were interested in. . . .they like reading but there was no books at all in their flat. . . So, I took them to join the library. . .I offered to go shopping with them, and when I went for the next visit, they’d been themselves. So that was quite a big thing. . . because they’d not been doing anything. (Val)

The romantic genre can be characterised by a series of trials and Alex needed to negotiate several during a cooking intervention with a service user. They initially had no money for ingredients and no kettle to make tea. On a subsequent visit, they cook pasta. . .

They didn’t have a lid for the pan or a sieve or a colander so there is no way they could drain the pasta. So (laughs) I had to use like a side plate and I said them don’t do this normally, but. . .I want to have an end product. . . it was so nice to see them so happy because they’d obviously felt like they had achieved something. (Alex)

Alex’s perseverance is rewarded when the service user successfully completes the task.

Eli identified a more occupation-centred activity for the service user:

Occupational therapy, it was . . .doing exercises with their upper limb, and everything in my head was saying this is not occupational therapy. . . .they were just getting a bit bored. . . I said. . .why don’t we see if you can eat some breakfast?. . . their face like immediately lit up. . .they even said to me that is more meaningful to me. . .It was kind of a bit of like an a-hah moment where all the reasoning came together. (Eli)

### Travel

Seven participants used the travel genre. This genre emphasises differences and contrasts, success and failure, and the individual is static and unchanging (Bakhtin, 1986). It is a genre without emergence and development, the individual’s existing perspective remains unchanged (Bandlamudi, 1999).

Several participants used this genre when describing reasoning in acute physical settings. Sam contrasted learning at university with their experience on placement:

That was a real. . .shock . . .and gulf, . . .that split between what we had been learning at university. . . looking at all the person, environment, occupation . . .and then going into the first placement. . . .this doesn’t look like occupational therapy. . .just getting on and off the toilet. (Sam)

The pace of an acute setting impacted reasoning:

Your narrative. . ., that was a lot more stifled. . . that’s where I struggled. . .The anxiety of the pace kind of made my clinical reasoning shut down. Even though afterwards I knew what I was seeing and what needed to be done. (Jordan)

Quite overwhelming, and it was so fast that there wasn’t time to think, . . .kind of unsure about what the purpose of occupational therapy was, or why we were there to give out commodes. (Rowan)

Also, the fast-paced reasoning of an experienced therapist could be difficult to follow:

I went out with band six and seven’s. . .they were so fast, . . .they would go through their reasoning. But I think sometimes they would *miss out things* that they might think was obvious to discard. (Morgan)

Anxiety could inhibit reasoning:

Being all proper watched all the time . . .If I’m panicked, I can’t always attend to all the information around me and I can’t remember things that I’ve read. . ., or what I need to be looking for. . .I prefer working with service users on my own. . . .they don’t know all these clinical reasonings and things that are going on inside your mind. (Eli)

I was a little bit flustered. . .I just completely and utterly went blank. . ., I think, because. . . I did feel very nervous. (Val)

### Adventure

In this genre, the adventures the individual experiences shape their identity and there is transformation and change in the individual (Bakhtin, 1981: Bakhtin, 1984). Two participants used this genre when speaking about feedback on their reasoning from their educator:

Placement two. . . was a bit of a boot camp in SOAP notes. . . telling me that I was wrong all the time. . . .this is what they want the SOAP notes to look like. . .I still don’t write notes how they would have. . . My last educator said. . .you’re developing your own style. . .I can see where your reasoning’s coming from. . .Don’t think we could have had that without that openness in placement two. . . listening to my educator’s reasoning. . .looking at their SOAP notes and. . .adopting them. (Eli)

The adventure genre is associated with metamorphosis; a sequence of guilt, retribution, redemption and blessedness (Bakhtin, 1981).

For Eli, guilt was associated with always being wrong, then the retribution of a SOAP notes boot camp, followed by the redemption of improved notes after emulating their educator’s approach. Finally, the blessedness of positive feedback in another placement:

Everything I did was always ‘why did you do it like that?. . .That’s not right’. . . Always negative. . .It was my first time I’d ever been in a hospital setting, because I didn’t know certain things it was almost like a disappointment. . .made me feel. . .very isolated. . . .just wanted to pass the placement. . . buckled down and thought okay, just do this. . .I learnt to speed up. . .make my writing more concise. . .the third one it was okay. . .I got good feedback. (Frankie)

Frankie describes the guilt associated with disappointing their educator, the retribution of the educator’s negative feedback, the redemption that came with improved notes, and finally the blessedness of skills for future placements.

### Carnival

Two participants used this genre when discussing the need to manage an unpredictable situation and reason at the moment:

They were like ‘. . .I really wanna butter this bread and. . .cut up some tomatoes and cucumber’. . . .I thought, mmm, I’m not so sure if this is going to work but just being like a student, I thought I’m just going to go with it [laughs]. . . .my reasoning wasn’t. . .in the moment. . .they were cutting it like with a knife going towards their hand, . . .had to stop them, and think. . .a lot of in the moment chopping and changing, pardon the pun (both laugh). . . .they were almost like my guinea pig. . . I was experimenting. (Eli)

The OCAIRS assessment. . .was impossible to do [clicks fingers] because the client was like floridly psychotic. . . would not respond to any of my questions and would just start talking about UFO’s, . . .They took the sheet off me. . . started writing things on it and drawing pictures. . .it was completely out of my control, and I didn’t know how to manage it really except. . . not being authoritarian. . .not trying to restrict. (Jules)

In this genre, hierarchical barriers between people are removed (Bakhtin, 1984). Both participants experienced a momentary loss of authority when the patient took control. This genre mixes the serious and the comic and relies on free invention (Bakhtin, 1984). Eli mixes the serious risk of injury with a comic chopping pun and mentions experimenting with their reasoning. The suspension of restriction in this genre (Bakhtin, 1984) is evident in Jule’s description of being ‘out of control’ and ‘trying not to restrict’.

The dialogical methodology in this study facilitated the exploration of emotion and Table 2 illustrates some of the emotions identified in relation to the participant’s reasoning.

**Table 2.** Emotions identified in relation to the different genres.

Genre	Emotions identified
Bildungsroman	Determination to persevere when reasoning was not easy. Illumination and amazement as reasoning developed. Feeling supported in their reasoning. Thankfulness for time and space to reason. Feelings of confidence in their reasoning as it developed
Romance	Empathy. Passion for person-centred and occupation-centred values. Feelings of beneficence (desire to do the best they could for the service user). The satisfaction gained by opportunities to enact their values
Travel	Disorientation. Feeling overwhelmed, challenged, pressured and stifled. Anxiety
Adventure	Determination, openness, independence. Feeling challenged. Sad, isolated and a disappointment to their educator. Pride in their achievements
Carnival	Bravery, freedom, impulsivity, irreverence, loss of control and feeling unrestricted. Isolation

## Discussion

This study aimed to explore how occupational therapy students experienced professional reasoning during their practice-based learning using a dialogical approach. Two major genres (Bildungsroman and romance) and three minor genres (travel, adventure and carnival) were used by participants when narrating their experiences.

The Bildungsroman can ‘simultaneously depict the unfolding of individual development and the enfolding of the individual into social institutions and roles’ Michelson (2012: 203). This genre was used to describe participants’ socialisation into the reasoning of the profession.

The participants experienced reasoning as complex and overwhelming. Novices find reasoning overwhelming due to difficulty identifying cues, a less organised knowledge base, limited pattern recognition and low automaticity (Carr and Shotwell, 2018). Exposing a student to varied experiences reduces opportunities for repetition and pattern acquisition. Pattern recognition helps filter information and identify cues, making reasoning less overwhelming. Repetition versus variation needs to be finely judged, as variation and complexity are important for the development of advanced reasoning (King et al., 2008; Mattingly and Fleming, 1994). Repetition will be useful when students are establishing new skills and variation once they have a foundation to build on.

Novices need more time to reason (Carr and Shotwell, 2018), and providing autonomy allows students to reason at their own pace. Autonomy is associated with the need to reason in the moment and errors in reasoning can occur due to limited experience. Errors are inevitable when reasoning (Cronin and Graebe, 2018) and can be valuable learning opportunities. A safe place to make mistakes is important in the development of reasoning.

Some participants found SOAP (Subjective, Objective, Analysis, Plan) documentation scaffolded reasoning. A profession’s paradigm has specific terminology and an approach to decision-making (Simpson and Cox, 2019). SOAP notes make reasoning explicit allowing the practice educator to socialise the student into the language and reasoning of the profession. Occupational therapy conceptual practice models also scaffold reasoning, Turpin et al. (2024) suggest they provide a foundation for reasoning and assist in professional socialisation.

Transformative learning theory suggests we have a frame of reference (assumptions influencing our views) and with support, we can challenge assumptions and unquestioned values (Cranton, 2016). A practice educator explaining their reasoning exposes students to an alternative view. Learning to reason about risk was transformative for some participants. Their inexperience meant they could be risk averse. A loss of idealism is characteristic of the Bildungsroman (Bakhtin, 1986), the participants needed to abandon the idea of eliminating all risk and balance risk with factors such as autonomy. The participants did not always consider context and use future-orientated conditional reasoning to anticipate potential adverse consequences of attempts to reduce risk.

Occupational therapy has a long association with romanticism drawing on romantic ideals such as a belief in the transformative potential of engagement in creative activities for health and well-being (Turner and Alsop, 2015). The romantic genre involves the embodiment of values (Bakhtin, 1981), and occupation-centred and person-centred values informed the participant’s reasoning and contributed to the development of their professional identity. Sonday (2021: 49) defines professional identity as ‘the way a professional defines who they are and the way they choose to act and represent themselves’. Identity theorists recognise the interaction of both the interior psychological and exterior sociological aspects of identity as individuals consider how they are the same as and different from others (Hammack, 2015). Walder et al.’s (2022) scoping review of occupational therapy professional identity explores identity largely at the level of the profession rather than the individual. This indicates that the identity of the profession has been the focus of most literature to date.

Participants used narrative reasoning to inform a person-centred approach. Narrative reasoning enables the individualisation of interventions based on an understanding of the service user’s story and values (Mattingly and Fleming, 2019). Hamilton (2018) suggests that narrative reasoning is characteristic of expert practice. Whilst experience contributes to expertise, motivation and in-depth critical reflection are also important (King et al., 2008; Mattingly and Fleming, 1994). Narrative reasoning requires empathy (Cronin and Graebe, 2018) and the participants’ empathy facilitated engagement with the service user’s narrative. Arguably empathy is primarily a personality trait (Meshovic and Mirovic, 2019) and therefore not dependent on experience.

Institutional systems impose constraints on person-centred and occupation-centred practices. Institutional values can conflict with occupational therapy values and result in a narrowing of reasoning (Cronin and Graebe, 2018; Mattingly and Fleming, 1994). Development of professional identity involves both identifying with and differentiating oneself from others. When students are exposed to new values, they may accept, refine or reject these (Sarraf-Yardzi et al., 2021). To date, much of the literature focuses on the individual accepting and becoming socialised into the profession. However, the participants in this study resisted socialisation into values incongruent with their developing professional identity.

Clarke et al. (2014: 226) suggest that role-emerging placements provide an opportunity to construct an individual professional identity, in contrast to students ‘passive assimilation of ideas, values and practices of others on traditional placements’. The participants in this study were mainly discussing traditional practice-based learning but at times actively resisted assimilating others’ values. Identity is regulated by the organisation, and students need to negotiate how much of their professional identity (influenced by previous experiences) they can express (Fenton-O’Creevy et al., 2015). Role-emerging settings, without an established occupational therapy role, allow the development of an individual identity, with minimal identity regulation. However, they also provide fewer opportunities to align and differentiate professional identity with that of other occupational therapists. Understanding how your values are the same or different from others is an important aspect of becoming an autonomous, self-regulating, practitioner. Our contention is that traditional and role-emerging placements both offer good (but different) opportunities to construct a professional identity.

Occupational therapists can experience difficulties in maintaining an occupational focus in their practice. Di Thommaso et al. (2019) found newly qualified practitioners regarded a focus on occupation as a luxury, conforming to a workplace culture of impairment-focused practice. This narrowing of the occupational therapy role is concerning, as the occupation-centred practice expands professional reasoning and enhances professional identity (Turner and Knight, 2015). A strong professional identity contributes to resilience (Ashby et al., 2012) and can therefore facilitate retention, and graduates’ successful transition into the workplace. Difficulties in enacting person-centred and occupation-centred values may reduce resilience, impact retention and impair the development of professional identity. There is perhaps a tension between a system that values resilience and a well-formed professional identity but imposes constraints that inhibit the development of these.

Some participants used the travel genre to discuss their experience of reasoning in fast-paced acute physical settings which inhibited the enactment of their person-centred and occupation-centred values. Britton et al. (2016) identify the challenges of implementing occupation-centred practice and establishing an occupational therapist’s identity in these settings. The fast-paced nature of reasoning in acute settings could be experienced as overwhelming. In acute hospitals, reasoning is rapid and condensed, requiring focused questions (Britton et al., 2016). Focused questioning requires recognition of important cues. As novices find it difficult to determine which cues are important and reason slowly (Carr and Shotwell, 2018), a fast-paced setting can be challenging for students.

Practice placements can be anxiety-provoking and being observed can inhibit reasoning. Unintended consequences of observation can include anxiety about being assessed altering performance, mistakes increasing, and students being more task-orientated and less patient-centred (LaDonna et al., 2017). Providing autonomy, when appropriate, may reduce anxiety and facilitate the development of reasoning.

The adventure genre centres around transformation, from guilt, to retribution, to redemption, then blessedness (Bakhtin, 1981). Some participants used this genre to describe the guilt of disappointing their educators, the retribution of negative feedback and redemption when resilience enables them to meet expectations. They learned from the experience and were ‘blessed’ with reasoning skills to use in future placements. This transformation suggests that transformational learning is occurring. Transformational learning can be emotionally challenging and is aided by a supportive individual and environment (Cranton, 2016). Current models of practice education are based on cognitive and constructivist approaches with the educator facilitating learning, rather than behavioural approaches in which the student emulates their educator (Rodger et al., 2014). These participants needed to adapt to a behavioural approach and emulate their educator’s method of documenting reasoning. They took a strategic approach to pass the placement and were not committed to the approach being modelled. This is described as unengaged alignment in a community of practice, the required practice is performed without the individual aligning themselves with the community (Kubiack et al., 2015).

Qualities in a practice educator that facilitate learning include, a student-centred approach taking account of the student’s feelings and provision of balanced, positive and encouraging feedback (Rodger et al., 2014). These participants did not experience this; however, their resilience and emotional intelligence facilitated a positive outcome. Resilience involves the ability to adapt and control your feelings (Abbott, 2018). The participants adapted to emulate their educator’s reasoning and managed the emotions engendered by receiving feedback they experienced as negative. Both internal factors such as locus of control and external factors such as supportive supervision and coaching (Abbott, 2018) contribute to resilience. These participants relied on their inner resources.

In the carnival genre, there is a freeing from authority (Bakhtin, 1984). Sometimes when working autonomously, participants momentarily lost control of an assessment or intervention when they were unable to reason quickly when something unexpected happened. With a little time to reason they regained control. It was a useful learning experience, especially when an educator supported reflection on the multifactorial nature of the reasoning required. Occasionally, a participant did not receive feedback and support, and sometimes rather than emulating their educator’s reasoning, they disassociated themselves from it.

Learning to reason is not just a cognitive process, it is also an affective process. However, affect is rarely studied in relation to reasoning. Koufidis et al. (2020) found an affective component (uncertainty, frustration and powerlessness) when medical students learned to reason. This has some parallels with our findings in relation to the participants’ experiences of anxiety, frustration, confusion and feeling challenged and uncomfortable. Healey (2017) explored emotion management during occupational therapy students’ practice-based learning. They found that students attempted to hide emotions to appear professional. In our study, a large range of emotions were identified in relation to both the challenges and rewards of learning to reason (see Table 2). It is important to consider the emotionality associated with reasoning and not to see reasoning as a purely cognitive exercise. Our study adds to the sparse literature on occupational therapy students’ emotional experiences during practice-based learning.

Further research could explore emotionality and practice-based learning, the development of students and newly qualified practitioners’ professional identity. Also, reasoning in acute physical contexts.

### Strengths and limitations

The dialogical approach elicited a rich representation of the student’s experiences, facilitating the exploration of emotion and identity. This approach required a significant investment of time to develop the knowledge and skills required for data analysis. As this was the first author’s PhD study, this was possible, but this would need consideration if adopting this approach for the first time. The open research question and unstructured nature of the interview meant a considerable amount of relevant data was obtained, this together with the decision to include both major and minor genres has limited the depth of the discussion. A smaller sample may have increased the depth in which the findings could be explored. However, the openness of the research question and the unstructured nature of the interview were important in foregrounding the participants’ narratives. Also, the inclusion of minor genres allowed a wider variety of experiences to be represented. This was important as the minor genres illustrated some experiences that were challenging for the participants.

## Conclusion

To our knowledge, this is the first study to explore occupational therapy students’ experiences of reasoning during their practice-based learning. The findings illustrate that reasoning during practice-based learning is complex, multifactorial and contextually influenced. Reasoning is influenced by the student’s professional identity as well as socially constructed through a process of professional socialisation. Our findings illustrate the role of empathy, narrative and emotion in students’ reasoning and the conflicts they can experience with educators’ and institutional expectations when being person and occupation-centred in their reasoning. Consequently, they can struggle to reconcile what they see in practice with their developing professional identity. Practice educators should support students to reflect on their reasoning and discuss the emotions experienced. They could share their strategies for remaining person and occupation centred in their practice. Universities should prepare educators to support students with the emotionality associated with reasoning and bridging the theory-practice gap. The curriculum should include opportunities for students to voice the emotionality associated with reasoning and explore the pragmatic constraints of real-world practice.

**Key findings**

- Reasoning is a means of enacting professional identity, and of being professionally socialised.
- Narrative, empathy, person and occupation-centredness inform reasoning but can conflict with institutional/educator demands.
- Reasoning is an emotional as well as a cognitive process.

**What the study has added**

Professional reasoning is a means of affirming and negotiating a professional identity, and of being socialised into the profession. Students can experience conflicts with institutional and educator demands when using person and occupation-centred reasoning.
